# Co-Creating an Occupational Health Intervention within the Construction Industry in Sweden: Stakeholder Perceptions of the Process and Output

**DOI:** 10.3390/ijerph182412872

**Published:** 2021-12-07

**Authors:** Emma Cedstrand, Helle Mølsted Alvesson, Hanna Augustsson, Theo Bodin, Erika Bodin, Anna Nyberg, Gun Johansson

**Affiliations:** 1 Unit of Occupational Medicine, Institute for Environmental Medicine, Karolinska Institutet, 171 77 Stockholm, Sweden; theo.bodin@ki.se (T.B.); bodin.erika@gmail.com (E.B.); anna.nyberg@pubcare.uu.se (A.N.); Gun.johansson@ki.se (G.J.); 2 Department of Global Public Health, Karolinska Institutet, 171 77 Stockholm, Sweden; helle.molsted-alvesson@ki.se; 3 Procome Research Group, Medical Management Centre, Department of Learning, Informatics, Management and Ethics, Karolinska Institutet, 171 77 Stockholm, Sweden; hanna.augustsson@ki.se; 4 Center of Occupational and Environmental Medicine, Stockholm Region, 113 65 Stockholm, Sweden; 5 Department of Public Health and Caring Sciences, Uppsala University, 751 22 Uppsala, Sweden

**Keywords:** co-creation, occupational health intervention, stress, psychosocial work environment, implementation

## Abstract

One way to prevent work-related stress, is to implement primary occupational health interventions aimed at improving the psychosocial work environment. However, such interventions have shown a limited effect, often due to implementation failure and poor contextual fit. Co-creation, where researchers, together with end-users and other relevant stakeholders, develop the intervention is increasingly encouraged. However, few studies have evaluated the effects of co-created interventions, and participants’ experience of the co-creation process. This is one of the first studies evaluating stakeholder perceptions of co-creating an occupational health intervention. We applied a thematic analysis, with data from 12 semi-structured interviews with stakeholders involved in the co-creation. Our results show that the respondents, in general, were satisfied with engaging in the co-creation, and they reported an increased awareness regarding risk factors of stress and how these should be handled. Additionally, the respondents described trust in the intervention activities and a good fit into the context. The study indicates that co-creating occupational health interventions can enhance the implementation and the contextual fit.

## 1. Introduction

Stress is a rising global health problem and has been classified as the health epidemic of the 21st century by the World Health Organization. A poor psychosocial work environment with, for example, low control in combination with high demands is a suggested cause of stress-related ill-health [1,2]. Hence, improving the psychosocial work environment may be one way to deal with this problem. However, primary organizational interventions to improve the psychosocial work environment and mental health have shown a limited effect [3,4,5]. One explanation for this is that the intervention was not carried out as intended, i.e., the implementation fidelity was low [6,7,8]. Suggested reasons for low implementation fidelity are poor contextual fit of the intervention [9,10] and lack of readiness for change [11,12]. To develop more efficient interventions, co-creation, where researchers, together with end-users and relevant stakeholders, develop the intervention, is increasingly encouraged in public health [13,14].

One aim of co-creating interventions is to enhance contextual fit [10,15], which involves tailoring the intervention to the workplace context because interventions need to be responsive to the participants they serve [16]. Instead of only relying on theory to guide the creation of interventions, local knowledge regarding structures and values must be utilized [10]. Another aim of co-creation is to enhance readiness for change, which is also vital for behavior change interventions within an organization to be successfully implemented [12]. High readiness for change postulates that the intervention participants are willing and able to make the required changes [17,18]. Participants need to perceive the intervention activities as relevant and corresponding to the problems raised to enhance their engagement. Hence, their willingness to change will be high.

Co-creating interventions within public health mean that researchers collaborate with end-users such as patients, schoolchildren, or employees and other non-academic stakeholders such as service providers, policymakers, and managers [15,19,20,21]. The collaboration can include the development of the agenda, design and implementation of the intervention, and interpretation and dissemination of the findings. Leask et al. [15] presented one framework for co-creating and evaluating public health interventions [15]. They define co-creation as “collaborative public health intervention development by academics working alongside other stakeholders” ([15], p. 2). Because the implementation process is important for the success of an intervention, i.e., the intended outcomes are achieved [4,22], we will build on this definition and add implementation. Thus, we define co-creation as “collaborative public health intervention and implementation development by academics working alongside other stakeholders”.

Empirically, co-creation and co-production have been interchangeable concepts [23], with co-design as a third option applied. Moreover, within occupational health intervention research, participatory design (PD), i.e., allowing employees to influence what and how to change the work environment [24], is commonly used [25]. Co-creation and PD could easily be mixed up as they have many commonalities; for example, they aim to ensure contextual fit and readiness for change [15,24,26]. However, there is at least one crucial difference. We suggest that co-creating an occupational health intervention facilitates power-sharing and the involvement of end-users and relevant stakeholders, without forcing the intervention activities to be participatory. Distinguishing different types of participatory approaches could enable a better understanding of which types of participation are most effective in improving the work environment and, ultimately, mental health.

Moreover, to facilitate replicability and enhance comparison between different co-creation processes, it is essential to clarify the end-users’ and other stakeholders’ roles [15,24]. The conceptual model of participation in work environment interventions [24] specifies the aspects of *content*, *process*, *directedness,* and *goal*. Content refers to what is to be changed, for example, which kind of working conditions are targeted. The process reflects how the content is delivered and can be seen as the implementation of the intervention. Directedness refers to whether all end-users are involved or if elected or appointed representatives are used. It should be clarified whether the goal appears to have a meaning in and of itself or if the participatory approach is a means to reach other goals. Such goals might be the fit of the intervention into the workplace, readiness for change, and buy-in and support among managers. The conceptual model of participation is not described concerning co-creation [24]. However, we argue that the model is helpful to clarify the participatory characteristics of a co-creation process.

Although many benefits of involving stakeholders in developing the intervention and implementation have been proposed [27], few studies have evaluated the effects of co-created interventions [15,19] and participants’ experience of the co-creation process. This study is part of a larger project involving developing, implementing, and evaluating an occupational health intervention to improve the psychosocial working conditions and decrease stress within a construction company in Sweden [28]. In this study, we seek to answer the following research question: What were the participants’ (a) experiences of the co-creation and learning processes and (b) perceptions of the intervention activities and implementation strategy?

## 2. Materials and Methods

The reporting of the methods has been guided by the criteria for reporting qualitative research (COREQ) guidelines [29]. The Swedish Ethical Review Authority (2019-02662) has granted ethical approval.

### 2.1. Study Setting and Design

The study was conducted within the construction industry in Sweden, which occupies around 300,000 individuals and is male-dominated. We enrolled one large well-established Swedish multinational company in the study. This qualitative study was nested within a controlled trial of an occupational health intervention to improve the psychosocial work environment and decrease stress. Details of the controlled trial are published elsewhere [28]. Two regions in Sweden participated in the study, one of which was the target group of the co-creation process. The gender distribution in this region was 80% men and 20% women. The other region served as the control group.

### 2.2. The Co-Creation

The goals of the co-creation were to (a) define goals, intervention activities, and implementation strategies, (b) enhance readiness for change and tailor the intervention into the context, (c) knowledge exchange, and (d) improve the dissemination of findings. We applied co-creation as a means of achieving high implementation fidelity, i.e., to ensure the intervention was delivered as intended and the end-users adhered to it [28]. We used appointed representatives, i.e., members of the Health and Safety advisory Board (HSB), explained below. The members had high participation over the content as they decided which psychosocial working conditions to target and how they could be changed (intervention activities). All appointed representatives were also allowed to influence the implementation strategies, even if the highest management team decided the form.

**Figure 1:**
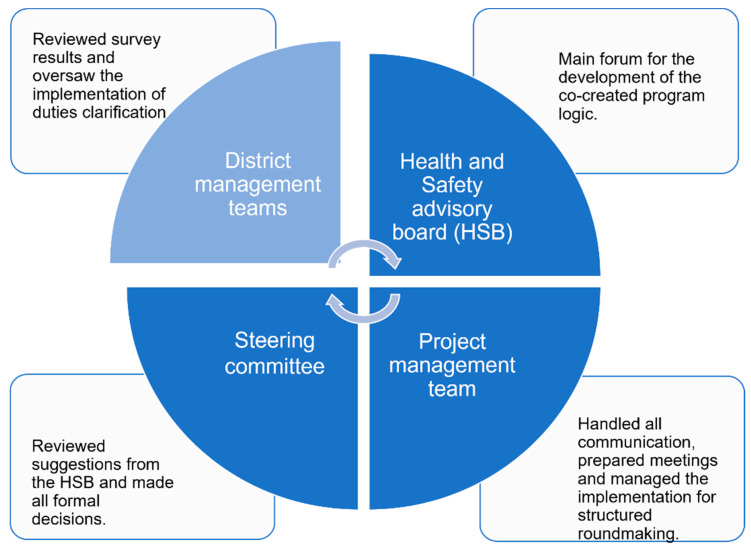
Groups involved in the co-creation process and their different roles.

The first author led the co-creation of the outcomes, intervention activities, and implementation strategies (program logic), together with the members of the HSB. See Figure 1 for the groups involved in the co-creation and their different roles. The HSB was an already existing group with representatives from all levels and districts within the organization. Thus, it was well suited for the co-creation. All workshops were held in regular meetings; thus, we inserted new content into existing structures. The members of the HSB were actively engaged in contributing to all workshops. The project management team consisted of E.C., who is a chartered psychologist working with industrial and organizational psychology, the HR representative, the manager, of Health and Safety, the Business Development Manager, and Manager of Operations. The fifth author (E.B.) was involved in the project from January 2020 until February 2021 and was part of the project management team. She also conducted interviews with the first-line managers and safety representatives in May/June 2020. E.B. has a Master of Arts in Psychology and has worked with HR questions for many years. E.C. led all meetings with the HSB, the district management teams, and the highest management team.

The co-creation process was developed in three waves, starting with a needs assessment. The researchers performed interviews, which informed the construction of the questionnaire. The interviews and the questionnaire constituted the needs assessment, which the researchers administered. For a complete list of co-creation activities, see Table 1.

The second wave included meetings with the HSB to co-create the program logic and implementation strategy and meetings with the project management team and the highest management team. The final wave comprised the implementation, including the Production Academy (described below) and meetings with all co-creation groups to monitor and follow up on the implementation process.

The overall aim of the co-creation project was to increase knowledge regarding the psychosocial work environment and how it could be improved. E.C. supplied information regarding well-known demands and resources within the psychosocial work environment to all groups (Figure 1) and discussed them concerning the survey results. The survey collected information regarding the following demands: quantitative demands, work pace, role conflict, job insecurity, and work-life conflict, which came to be called wearying factors. Further, the survey collected information regarding the following resources: role clarity, social support from supervisor, work engagement, and influence, which came to be called buffering factors. Information on stress was collected as an outcome.

Results from the needs assessment demonstrated unfavorable levels of stress, quantitative demands, and role clarity. Therefore, we targeted them as the main goals of the intervention. To improve these factors, structured roundmaking, (i.e., to make structured and collective planning for all elements of the production) and duties clarification were chosen as intervention activities. For detailed information, see the study protocol [28].

At a workshop with the HSB, the behavior change wheel (BCW) [30] was introduced.

The BCW is a framework to facilitate the design and description of behavior change interventions to enhance the implementation. The BCW is a compilation based on 19 frameworks classifying behavioral change interventions.

The BCW consists of three parts or layers. The COM-B model represents the inner layer and stipulates that people need capability (C), opportunity (O), and motivation (M) to perform a behavior (B). The middle layer describes nine intervention functions or strategies applied to facilitate the behavior change. Examples of these functions are Education, Training, and Modelling. The last part is the outer policy layer, which comprises seven policy types, enabling the nine intervention functions to occur. The BCW has been used to guide intervention design in a various health care settings, for example, smoking cessation [31] and alcohol reduction [32].

Our aim with introducing the BCW to the HSB was to increase the knowledge regarding possible aspects, i.e., capability, opportunity, and motivation, affecting behavior change. Additionally, the nine intervention strategies were described to display the range of options available to promote behavior change. After explaining the two inner layers of the BCW, we analyzed hindering and facilitating factors taking the different parts of the COM-B model into account. Examples of hindering factors related to capability were lack of knowledge regarding structured roundmaking (intervention) and lack of understanding of how to practice it. Once the hindering factors were identified, the group members were encouraged to develop strategies to meet the recognized needs. For example, Education and Modelling were seen as appropriate strategies. After the workshop with the HSB, the project management team had additional discussions to finalize the implementation strategy. Four strategies were chosen: (1) identifying early adopters of the intervention activities, (2) shadowing other experts, (3) visiting other sites, and (4) creating a learning collaborative. To enable comparison with other studies, our strategies correspond with the ones described by Powell et al. [33].

The implementation support was called the “Production Academy”. At the meetings, the managers of the construction projects were allowed to discuss and plan their development regarding structured roundmaking. They were also supposed to share good examples and visit other projects to pick up good examples of implementing structured roundmaking. However, due to the COVID-19 situation, most of the meetings were held online, which impeded the construction project members from fully engaging in sharing good examples and visiting each other’s projects. It also led the highest management team to only enroll the four largest projects in the implementation support instead of all eligible projects. Production teams eligible for the implementation support were the ones at the right stage of production, meaning that teams at the very start or the end of a project were omitted. The researchers were not involved in the delivery of the implementation support. The unit of analysis of this study was individuals taking part in one or more of the co-creation activities.

### 2.3. Participants for the Interviews for the Research Study

We based the study’s sampling method on the assumption that respondents’ perceptions could differ depending on their role in the company, the co-creation process, and gender. Therefore, we applied the principles of maximum variation during the purposive sampling process to select participants from all levels within the company, from the different co-creation groups (Figure 1), and of a different gender. Once the participants were identified, the HR representative contacted them, asking for approval for the researchers to contact them. Thirteen persons accepted to be interviewed, and two declined, one due to time constraints and one because he felt he had not participated enough in the co-creation meetings. One person who accepted to be interviewed later canceled the meeting due to time constraints. In total, eight men and four women participated. See Table 2 for a full list of the study participants. All respondents were given written information and gave written consent to participate.

### 2.4. Data Collection

Twelve semi-structured interviews were conducted in December 2020 and January 2021. They were held online due to COVID-19 and lasted, on average, 37 min (range 19–55 min). E.B. conducted all interviews as she had not been part of the project’s setup, nor had she participated in the workshops with the HSB or in the feedback meetings with the district management teams. The interview guide was informed by the suggestions of Leask et al. [15] regarding the three areas to evaluate within co-creation projects: (1) satisfaction with engaging in the process, (2) perceived knowledge, and (3) skill development. We added questions on perceptions of the intervention activities and implementation support.

### 2.5. Data Analysis

We applied a thematic analysis guided by the stages recommended by Braun and Clarke [34], described below. All interviews were recorded and transcribed verbatim by a professional transcription firm. In the first stage [34], EC. and E.B. familiarized themselves with the data by reading the transcriptions and taking notes. In the second stage, E.C. made an initial round of coding in NVivo 12. The codes were organized according to the three areas in Leask et al. [15]; (1) satisfaction with engaging in the process, (2) perceived knowledge, and (3) skill development. In the next step, E.C. and E.B. separately coded the data to obtain a comprehensive and nuanced coding of the data rather than seeking consensus [35]. The codes were discussed with HMA and G.J., after which changes were made. In the searching for themes, i.e., the third stage described by Braun and Clarke [34], it became evident that the three areas in Leask did not correspond well with the participants’ perceptions. Hence, the developed codes of these perceptions remained; however, we applied a data-driven approach in the development of themes. Steps four and five, reviewing, defining, and naming themes, were combined and carried out in an iterative process with feedback from HMA and G.J. Lastly, all authors discussed the results, and we conducted the final changes.

## 3. Results

**Figure 2:**
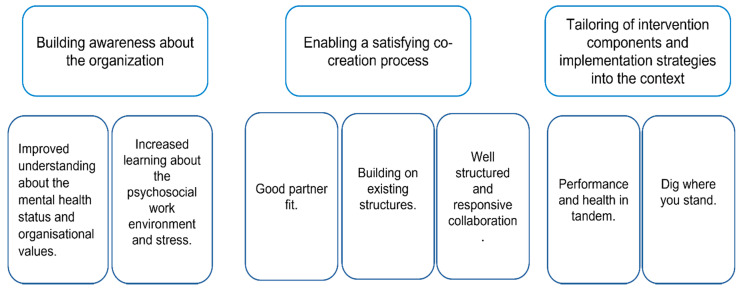
Themes and sub-themes describing respondents’ experiences of the co-creation and learning processes and perceptions of the intervention activities and implementation strategy.

Three themes representing the respondents’ perceptions of the co-creation process were constructed from the data: (1) Building awareness about the organization, (2) Enabling a satisfying co-creation process, (3) Tailoring of intervention activities and implementation strategies into the context. See Figure 2 for themes and sub-themes.

### 3.1. Building Awareness about the Organization

Through the co-creation process, respondents described gaining insights and increased learning, categorized into two sub-themes. The first one reflects improved understanding of the organization’s status, while sub-theme two reflects learning about health theories of stress and how these can be applied.

#### 3.1.1. Improved Understanding of the Mental Health Status and Organizational Values

Respondents explained that the needs assessment results increased their understanding of the mental health status among the employees. One erudition they spoke highly of was that the first-line managers reported the most stress when comparing different roles. Before the project, everyone assumed that the production managers were the ones struggling the most with stress. This awareness was perceived as applicable as it helped them target the right group for the intervention activities and valuable information for future health-promoting measures. Moreover, the respondents mentioned that the discussions during the project resulted in new insights and awareness regarding company values, such as mental health promotion. One blue-collar worker framed it like this:

*“One thing I take with me is that my company is actually investing in these matters (mental health). It is valuable to see that there is actually an ongoing work within the company, and even higher up in the chain, they take these matters seriously and engage in what we are going to work with. So, it feels good.”*

Safety representative (6)

#### 3.1.2. Increased Learning about the Psychosocial Work Environment and Stress

It was mentioned that working with the researchers contributed to a more profound understanding of different aspects of the psychosocial work environment and its relationship to stress. The respondents described that, even though the concept of a psychosocial work environment was not new to them, it was somewhat difficult to grasp and fully understand its practical meaning. The concept was operationalized and applied to their organizational context through the survey, which contributed to their greater understanding. One participant described this in terms of getting a new and mutual language for these types of questions:

*“So, the region has gotten a lot out of this (the project) I would say, and we as individuals have got a language, we had never used the word role clarity before, but I have done so now. Yes, so we have learned and gotten a lot out of this.”*

Manager CCF/GF (12)

They also reported that the information regarding what came to be called “buffering” (resources) and “wearying” (demands) factors in the psychosocial work environment were valuable. Several respondents mentioned these concepts as an example of an increased understanding of how the psychosocial work environment could impact stress. More resources were mentioned, and increased influence and how superior support could balance high quantitative demands and prevent stress was cited as an example of new knowledge.

### 3.2. Enabling a Satisfying Co-Creation Process

The respondents were generally satisfied with the co-creation process, and their experiences revealed three sub-themes, which seem to have contributed to their satisfaction. The first two sub-themes can be recognized as preconditions, while the third sub-theme adheres to the quality of the collaboration between the researchers and the involved stakeholders from the organization.

#### 3.2.1. Good Partner Fit

One precondition enhancing the co-creation process was the mutual expectations of how the project should be carried out. Both parties preferred a co-creation process with shared power, which is one goal of co-creation. The highest management team made it clear that if they were to engage the organization in a research project, it had to be collaborative. They expressed the desire that they did not want an off-the-shelf concept. Instead, they demanded involvement and continuous feedback. However, at the same time, they accepted that the researchers would set some ground rules to safeguard the quality of the research. The respondents expressed that they were satisfied with the balance between them and the researchers regarding sharing responsibilities and ownership throughout the whole process. They perceived the researchers as contributors to the framework and sources of expertise, while the organization had the authority to decide several questions. One participant mentioned an example of this:

*“We have tried to take responsibility and we have, among other things, influenced the questionnaire in a way which suited us. So, we have felt an ownership, I hope not too much.”*

Manager CCF/GF (5)

#### 3.2.2. Building on Existing Formal Structures

All groups involved in the co-creation, except one (project management team), existed before the project. Hence, only minor adaptions were needed to form the co-creation structure. The respondents perceived that the co-creation process was easy to fit into existing structures, specifically due to the already existing HSB. As this group already existed, content regarding the psychosocial work environment and stress could easily be integrated. However, a limitation within this group was that the blue-collar workers were underrepresented, which participants from different levels (managers and blue-collar workers) commented on. One blue-collar worker explicitly expressed a desire to involve this group to a greater extent, while the employer representatives reflected on whether it would have been beneficial to have more blue-collar workers involved. Even though most comments on the co-creation structure were positive, all members in the project management team mentioned that the roles and responsibilities within this group could have been clarified. The role uncertainty was thought to have slowed down the pace of the implementation, even if losing pace was primarily explained to be caused by the pandemic.

#### 3.2.3. Well-Structured and Responsive Collaboration

All respondents expressed that they were satisfied with the collaboration in general and, more specifically, they saw the overall structure and the implementation design as positive. They perceived the project objectives as straightforward, and they recognized the logic model as a helpful tool to identify outcomes and essential intervention activities. Some respondents mentioned wanting to try the model again with other issues. Efficient use of time was also a recurring comment in the interviews. Further, several respondents expressed being satisfied with the meeting set up and that the researchers participated in many different types of constellations. Several respondents also expressed contentment with being listened to and said that they had the opportunity to have their voices heard.

### 3.3. Tailoring of Intervention Activities and Implementation Strategies into the Context

Involving different stakeholders and allowing the organization to decide the intervention activities and the implementation strategies appears to have enabled a good contextual fit. The first sub-theme concerns the intervention activities and the second relates to the implementation strategies.

#### 3.3.1. Performance and Health in Tandem

Respondents found the intervention activities concerning increased productivity and improved mental health (less stress) to be applicable, which seemed to have enhanced the trust in the intervention activities and the willingness to implement them. This can be seen as an example of tailoring of the intervention activities, not only to aim for improved health but also, at the same time, to contribute to the business objectives. One manager framed it accordingly:

*“I absolutely think that the intervention activities are fully relevant to work with, and I think it will lead to, well…better structure in the projects, which I think leads to better mental health as well.”*

Manager CCF/GF (2)

While one of the intervention activities (duties clarification) was perceived as meaningful and easy to grasp by everyone, the second intervention component (structured roundmaking) was associated with some reluctance from some respondents. They mainly described the vagueness of the concept structured roundmaking as challenging.

#### 3.3.2. Dig Where You Stand

Respondents perceived the implementation support (Production Academy) as relevant. They expressed faith in the basic logic, “dig where you stand,” with each project or individual setting up its own goals and moving towards a behavioral change at its own pace. Another example of the concept “dig where you stand” was the decision to enroll the projects in the academy depending on what stage they were in. Construction projects being at the very start, or at the end, were omitted from the enrolment. We did this to ensure relevance and feasibility for each construction project to implement the intervention activities, and this can be seen as an example of tailoring. The respondents mentioned that taking the production stage into account to tailor the implementation support was out of the ordinary when it comes to implementing new routines. One manager´s words concerning this were:

*“But I have faith in the model (implementation support) that we select projects which are at the right stage (of production) and that we coach them based on their goals rather than sending individuals on a course and then they should come back and change something. That´s what I believe in”.*

Manager CCF/GF (5)

## 4. Discussion

This intervention study from the construction industry in Sweden shows that the respondents were generally satisfied with engaging in the co-creation, mainly due to the straightforward structure and the high degree of involvement and ownership. The results reveal that the respondents’ awareness of risk factors and how these should be handled increased with time. Finally, the study implies that the involvement of different stakeholders regarding content and process yielded trust in the intervention activities and fostered a good fit into the context. Improved dissemination of findings, i.e., the fourth goal of the co-creation, was not discussed in the interviews because the implementation was still ongoing.

The use of co-creation by researchers, end-users, and other relevant stakeholders when developing public health interventions is increasingly encouraged [13,14]. However, much is to be explored because few studies have evaluated the experiences of the co-creation process or more distal outcomes such as improved health among end-users [19]. To our knowledge, this is the first study evaluating participants’ experience of co-creation in the development of an occupational health intervention and implementation. This study adds to the literature by describing how such a co-creation process can be designed and by displaying positive experiences of the co-creation and learning processes and effects on readiness for change and tailoring of intervention activities.

Two preconditions seem to have supported the positive experiences and fulfillment of the goals of the co-creation. The first was the possibility to integrate the project into existing structures such as the HSB. The participants in this group were appropriate representatives in the co-creation because they represented different levels and groups within the organization. Integrating interventions into existing structures has been stressed in another study [36] because it showed positive effects on employee outcomes. Further, it was also time-efficient to use already scheduled HSB-meetings to discuss questions related to the project. This was a way to make sure the involvement in the co-creation was not seen as a burden, which other studies [37,38] have shown is harmful to the outcomes of an intervention.

In the same way, we believe it is essential that participating in a co-creation process is feasible without competing with regular work tasks. The HSB can be seen as a structure for involvement, allowing its participants to influence all possible changes related to health and safety. This type of structure can be seen as the end goal of many participatory work environment interventions because it increases employee influence [24].

Perhaps, one piece of the puzzle for organizations to succeed in implementing work environment improvements is to have a structure like the HSB. Understanding whether such a structure can facilitate change through representative participation is within the scope of future research. Additionally, this prerequisite, i.e., that the organization had a structure for representative participation, limits our findings’ generalizability. In a setting without this type of structure, representatives would need to be elected or appointed, and a structure for how they could meet would need to be created.

The other possible enabling precondition was the mutual expectations to share the responsibility and ownership between the enrolled organization and the researchers. This was manifested in the agreement to include the co-created intervention activities in the business case, which ensured a senior management commitment over time. A similar study [39] evaluating participants´ experience of co-creating a collective leadership intervention for health-care teams likewise stressed the importance of attaining a genuine co-creation partnership. They further highlighted the importance of a responsive co-creation process, allowing all participants an equal opportunity to influence the output. Our results support the idea of equal involvement because respondents across all levels reported satisfaction with being listened to.

Besides the described enabling preconditions, the results suggest three facilitating strategies in the co-creation design supporting the positive experiences and fulfillment of the goals. First, the use of the program logic [40] to guide the process towards specifying goals and actions (intervention) appeared successful. The approach was perceived as comprehensible as it gave a good structure to the co-creation process. Further, respondents appreciated the emphasis on clarifying the goals first before making action plans. This was a method the respondents reported that they wanted to try again when implementing changes in other domains. Applying co-created program logic is an example of how participation over the content can be employed.

Second, our results imply that allowing influence over both the content and the process seemed to have enhanced the implementation. The need to identify efficient strategies to support the implementation of workplace interventions is stressed [22,41]. However, explicit implementation strategies are often left out when planning and evaluating participatory work environment interventions [24]. For example, Leask et al. [15] did not include implementation (process) in their definition of co-creation, and it is shown that allowing participation over the process is less common than allowing participation over the content in participatory work environment interventions [24].

Third, the results imply that the use of representatives instead of involving all end-users was adequate to tailor the intervention into the context and to enhance readiness for change. This is in line with previous research on participatory organizational-level interventions, where Mellor, et al. [42] showed that the involvement of trade unions (representatives) facilitated the implementation of the studied intervention. Further, Cedstrand et al. [39] showed that supplying work groups with appointed representatives fostered positive reactions and facilitated change in behaviors and work routines. Furthermore, they found that the intervention activities involving all end-users led to adverse reactions and resistance towards the intervention among end-users. Nevertheless, even if the use of representatives seemed adequate to enhance the implementation, the results suggest that the blue-collar workers could have been involved to a greater extent.

Finally, as mentioned above, defining the implementation strategy and involving end-users in the design appears rare [24]. One reason for this could be the blurred borders between intervention activities and implementation in participatory work environment interventions. An insight we made from this project was that the use of a co-creation process supported a distinction between the intervention and the implementation, which is important for two reasons. Firstly, to make sure the implementation strategy receives enough attention and is made explicit. Secondly, it enables the evaluation of both parts [9].

### 4.1. Strengths and Limitations

One limitation of this study is the possible recall bias among participants, as the interviews were carried out one year after most of the co-creation activities took place. This might have led to less nuanced answers from the informants. However, at the time of the interviews, the intervention activities were being implemented. Performing the interviews earlier would have hindered the informants from describing their perceptions of the implementation. Including more managers from the production in the study would have been beneficial because they were the main target group for the intervention activities. However, the respondents represent all co-creation groups (Figure 1) and different levels and functions in the organization; thus, we have obtained sufficient information. Finally, it is both a strength and a limitation that E.B. and E.C. were involved in the co-creation and conducted the interviews and the analysis. The strength lies in their in-depth understanding of the process and the context to which they could relate the collected information. The limitation lies in that this might have hampered informants from reporting negative co-creation experiences due to social desirability [43]. The less involved researcher (E.B) performed all the interviews to mitigate this risk.

### 4.2. Implications

In addition to Leask et al. [15], who emphasize the need of evaluating experiences among participants, we also emphasize the need to evaluate whether the goals (e.g., improved implementation) of the co-creation were fulfilled. It is important to increase knowledge on *if* and *how* the co-creation can contribute to better conditions for the implementation process. We also find it important to co-create not only the intervention activities but also the implementation strategies.

## 5. Conclusions

The goals of the co-creation were to (a) define goals, intervention activities, and implementation strategies; (b) enhance readiness for change and tailor the intervention into the context; (c) knowledge exchange; and (d) improve the dissemination of findings. The results imply that the facilitating implementation factors, i.e., enhanced readiness for change and tailoring the intervention into the context, seemed to have been fulfilled. Hence, applying co-creation can be a helpful method in developing occupational health interventions and facilitating the implementation. This study suggests that important factors to succeed with the co-creation are: (1) Establishing a genuine partnership with mutual expectations regarding responsibility and ownership, where the senior management engagement over time and equal involvement for all participants is stressed. (2) Offering a clear structure for the co-creation to make the process comprehensible. (3) Integrating the co-creation in already existing structures. (4) Defining in what way and to what degree end-users and other stakeholders should be involved and, if possible, using representatives to avoid making the co-creation a burden. We encourage future studies to investigate these factors further.

## Figures and Tables

**Table 1:** Number of activities included in the co-creation process and when in time they occurred.

Activity	2019			2020				2021			
	Q2	Q3	Q4	Q1	Q2	Q3	Q4	Q1	Q2	Q3	Q4
Meetings Health and Safety Advisory Board	1	2	2	1			1	1			
Meetings Highest management team	1	2	2	1			1	1			
Meetings Project management team	2	1	1	1	1			1			1
Needs assessment Interviews with employees and managers from all levels to inform the survey.	25										
Survey Questionnaire on psychosocial work factors and stress.	NA		B				Fu				Fu
Feedback meetings Results from the surveys, District management teams		3	3	3	3	3	3	3	2	2	2
Interviews First-line managers and safety representatives					10						
Implementation support meetings Production Academy (4 projects)							2	1	1		

Q = Quarter, NA = Needs assessment, B = Baseline, Fu = Follow up.

**Table 2:** Characteristics of the study participants.

Participant	Professional Role	Group Affiliation in the Co-Creation Project (Figure 1)
1	Core Corporate Functions and Group Functions (CCF/GF).	Project management team, HSB ^1^
2	Manager CCF/GF.	Project management team, HSB
3	Safety representative	HSB
4	Safety representative	HSB
5	Manager CCF/GF.	Project management team, HSB
6	Safety representative	HSB
7	Manager production	HSB
8	Manager production	Highest management team
9	Manager production	Highest management team
10	CCF/GF.	HSB
11	CCF/GF.	HSB
12	Manager CCF/GF.	Project management team, HSB

^1^ HSB—Health and Safety Advisory Board.

## Data Availability

The data presented in this study are available on request from the corresponding author. The data are not publicly available due to legal restrictions.
